# Optimization for the efficient recovery of poly(3-hydroxybutyrate) using the green solvent 1,3-dioxolane

**DOI:** 10.3389/fbioe.2022.1086636

**Published:** 2022-12-06

**Authors:** Chanakarn Wongmoon, Suchada Chanprateep Napathorn

**Affiliations:** ^1^ Programme in Biotechnology, Faculty of Science, Chulalongkorn University, Bangkok, Thailand; ^2^ Department of Microbiology, Faculty of Science, Chulalongkorn University, Bangkok, Thailand

**Keywords:** 1,3-dioxolane, poly(3-hydroxybutyrate), environmentally friendly solvent, green solvent, recovery, scale-up

## Abstract

In this study, a simple non-toxic recovery process of biodegradable poly(3-hydroxybutyrate) (PHB) using the green solvent 1,3-dioxolane and water was successfully developed. The critical parameters were optimized, and the process platform was scaled up from 2 ml to 1,000 ml for the efficient recovery of PHB. The physical parameters including continuous shaking, ultrasonication, extraction using the Soxhlet extractor, diluted 1,3-dioxolane, reused 1,3-dioxolane, and cell rupture by steam explosion prior to solvent extraction were carefully investigated. The results showed that continuous shaking played a major role in increasing the recovery efficiency during the scale-up process. The PHB extraction at 2 ml from dried cells at 80°C with 100 rpm of shaking speed for 5 h resulted in a recovery yield of 96.6 ± 0.1% with purity up to 99.1 ± 0.6% and that from wet cells under the same condition resulted in a recovery yield of 94.6 ± 4.8% and purity of 97.0 ± 0.1%. It should be noted that the PHB extracted from wet cells at room temperature with 150 rpm of shaking speed for 36 h resulted in a recovery yield of 93.5 ± 0.7% and purity of 97.7 ± 1.3% and had an M_W_ of 3.1×10^5^, M_N_ of 2.7×10^5^, and polydispersity index of 1.1. The direct scale-up process at 1,000 ml showed comparable results in purity, recovery yield, molecular weight distribution, thermal properties, and mechanical properties. The PHB extraction from dried cells gave the highest purity of 99.3 ± 0.5% and recovery of 94.0 ± 0.3%, whereas the PHB extraction from wet cells gave a purity of 90.3 ± 1.5% and recovery of 92.6 ± 1.0%. The novel recovery process showed its feasibility to be applied on an industrial scale.

## Graphical Abstract

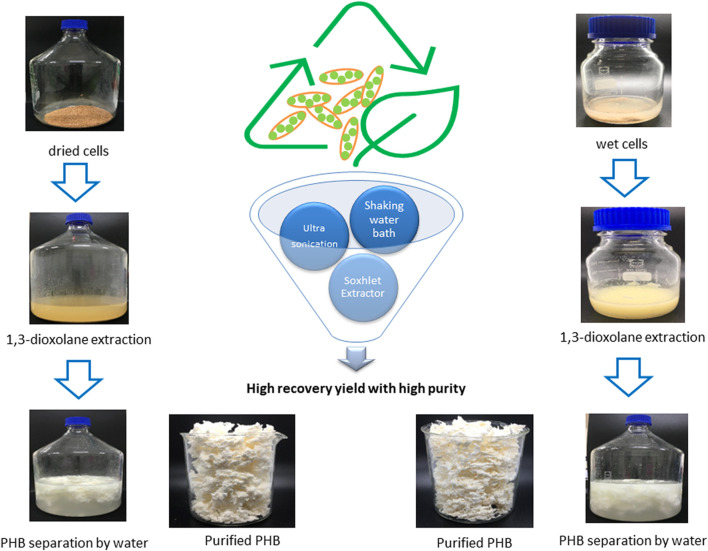

## Introduction

Microplastic pollution has been a major global concern as it is a severe threat to the environment, humans, and animals. Petrochemical-based plastics have superior advantages such as durability and resistance, but they become a hazardous waste after the end of use. This problem has called for alternative materials, such as polyhydroxyalkanoate (PHA), that are completely biodegradable in all environments. PHA is a class of microbial biopolymers comprising aliphatic esters of R-hydroxyalkanoic acids. The universal bottleneck involved in the commercialization of these biotechnological products is tightly associated with environmental concerns and cost-effective manufacturing. Although biodegradable PHA has the competitive advantage, it suffers from the high cost of raw materials and hazardous organic solvents used for the extraction from biomass. Recently, some scientists have attempted to utilize wastes that are associated with zero waste management in industry or municipality, such as utilization of waste effluents, which can also reduce pollutants and comply with sustainable management (Lai et al., 2022; Pei et al., 2022). In addition to reducing the production cost by finding less expensive substrates, downstream processes, including PHA extraction and purification, are still suffering from high operating cost and danger caused by toxic and flammable solvents. PHA is an intracellular energy storage compound accumulated in the form of PHA granules inside bacterial cells. The methods to extract PHA from bacterial cells can be performed in both dried cells and wet cells. The most well-known organic solvent that provides the best PHA solubility is chloroform, which is a carcinogenic and explosive reagent that is not suitable for large-scale downstream processes. Many researchers have tried to find cheap and environmentally safe solvents to replace the use of chloroform, such as sodium hypochlorite (Choi and Lee, 1997; Heinrich et al., 2012), linear and cyclic carbonates (Fiorese et al., 2009; Samorì et al., 2015; Fei et al., 2016), ethyl acetate (Riedel et al., 2013), and ionic liquids and supercritical fluids (Hampson and Ashby, 1999). Recently, several green extraction processes have been proposed. For instance, dimethyl carbonate (DMC) and ethanol as alternative solvents to chloroform and hexane have been reported with PHB yield values similar to or higher than those achieved by using chloroform (≥67%) (Mongili et al., 2021). PHB extraction using high-pressure CO_2_, enzymatic recovery, and 1,2-propylene carbonate has been demonstrated to achieve high yield and purity (Calleja Luque, 2020).

1,3-Dioxolane has been previously reported as a promising green solvent to extract PHB from both dried cells and wet cells (Yabueng and Napathorn, 2018). 1,3-Dioxolane is considered a green solvent as well as a reagent. It can be used as an alternative to dichloromethane, dichloroethane, and methyl ethyl ketone in neutral or basic reaction conditions or as a replacement of THF and dimethyl sulfoxide. It is used in the polymer industry as a copolymerizing agent and in the paint industry as a substitute for toluene and xylene (Moscoso et al., 2014). The copolymers of l-lactide with 1,3-dioxolane were reported to be used for the microfluidics synthesis of antimicrobial microcarriers loaded with quercetin, which is a natural compound that exhibits anticancer, antibacterial, and antiviral properties. Copolymers of l-lactide and 1,3-dioxolane are amorphous and exhibit enhanced degradation under acidic conditions (Kost et al., 2022). 1,3-Dioxolane derivatives are recognized as important motifs for the construction of numerous pharmacologically active molecules as antivirals, antifungals, anti-HIV molecules, and adrenoreceptor antagonists (Ram et al., 2019). The major advantage of 1,3-dioxolane over chlorinated solvents is that it is not carcinogenic or toxic to humans. 1,3-Dioxolane is a highly flammable liquid, with the determined flashpoint of ≤ 2.5°C and the boiling point of 76°C. 1,3-Dioxolane does not form flammable gases in contact with water and has no pyrophoric properties. The self-ignition temperature of 1,3-dioxolane is 250°C. Based on these data, 1,3-dioxolane is classified as a flammable liquid (cat. 2) (European Chemicals Agency, 2022).

Here, the authors put hard effort for the development of a chlorine solvent-free process for the extraction and recovery of PHB from *Cupriavidus necator* strain A-04. This will be a model for a novel recovery process based on water-miscible solvent systems. In the previous study, the extraction scale was reported with 2 ml for PHB extraction from dried cells at 80°C for 6 h in 1,3-dioxolane. Then, water was used for the phase separation of the PHB film from 1,3-dioxolane solution, which resulted in the recovery yield of 92.7 ± 1.4% and purity of up to 97.9 ± 1.8%. In this study, the factors affecting the PHB extraction efficiency using 1,3-dioxolane were reported. The authors successfully scaled up the extraction process from 2 ml to 40 ml and 1,000 ml and maintained the recovery as high as 94.1 ± 0.4% and purity up to 99.3 ± 0.5%.

## Materials and methods

### PHB-producing strain

*Cupriavidus necator* strain A-04 and an industrial strain *C. necator* H16 (as a control) were used in this study. The experiments were performed in a biosafety level 2 laboratory by the principal investigator and researchers who had undergone biosafety training.

### Preparation of wet cells and dried cells containing PHB granules in a 5 L bioreactor

The wet cells and dried cells containing intracellular PHB granules were obtained by batch cultivation in a 5 L bioreactor (MDL500, B.E. Marubishi Co., Ltd., Tokyo, Japan). The preculture was prepared in 500 ml Erlenmeyer flasks containing 100 ml of the preculture medium as described previously (Yabueng and Napathorn, 2018). The cells were harvested by centrifugation, washed twice with sterile 0.85% sodium chloride solution to remove the remaining nitrogen source from the preculture medium, and resuspended in 100 ml of 0.85% sodium chloride solution. The resuspended cells were inoculated into a production medium consisting of 5.8 g/L K_2_HPO_4_, 3.7 g/L KH_2_PO_4_, 0.12 g/l MgSO_4_⋅7H_2_O, 5 g/l sodium citrate, and 1 ml of trace element solution (1.67 g/l CaCl_2_⋅2H_2_O, 2.78 g/l ZnSO_4_⋅7H_2_O, 0.29 g/l FeSO_4_⋅7H_2_O, 1.98 g/l MnCl_2_⋅4H_2_O, 0.17 g/l CuCl_2_⋅2H_2_O). The dissolved oxygen (DO) and pH were monitored using a DO electrode (InPro6800/12/220, Mettler-Toledo GmbH, Urdorf, Switzerland) and a pH electrode (InPro3253i/SG/325, Mettler-Toledo GmbH, Urdorf, Switzerland), respectively. The set-point of the pH value was maintained at 7.0 ± 0.2 by automatically adding 2 M NaOH or 2 M H_2_SO_4_ solution with a pH controller (LABO-CONTROLLER MDL-6C, B. E. Marubishi Co., Ltd.). The working volume of the batch cultures was 3 L, the temperature was 30°C, the agitation speed was set at 500 rpm, and the air flow rate was 0.5 vvm. Fructose (20 g/L) was used as a carbon source, and ammonium sulfate was used as a nitrogen source. The carbon-to-nitrogen ratio was 200.

After 72 h of cultivation, the cells were harvested by centrifugation (10,160× *g*, 20 min, 4°C) and then washed twice with sterile 0.85% sodium chloride solution. Aliquots of cell suspension were used to prepare wet cells and dried cells. Wet cells were used as aliquots of fresh cells obtained directly from the washing steps. Dried cells were aliquots of fresh cells that were dried at 60°C for 24 h.

### Comparison of PHB extraction efficiency between *C. necator* strain A-04 and *C. necator* H16

A suspension of 0.1 g dried cells harboring PHB granules which was equal to 0.27 g wet cells of *C. necator* strain A-04 and *C. necator* H16 in 2 ml of 1,3-dioxolane was prepared in screw-capped borosilicate-glass test tubes. Parallel extraction with chloroform was performed as a control experiment. PHB was extracted from dried cells by incubating samples at 80°C for 6 h (Yabueng and Napathorn, 2018). The samples were mixed using a vortex mixer every 30 min. Next, PHB was separated from the solution by adding 3 volumes of water. Then, purified PHB floating on the surface of the solution was separated by centrifugation at 4,620× *g* for 5 min at room temperature. The purified PHB was washed twice with water and dried overnight in an oven at 60°C and kept in a vacuum desiccator. The purified PHB was analyzed in terms of percentage purity and recovery by gas chromatography.

### Extraction of PHB

#### PHB extraction using diluted 1,3-dioxolane

To reduce the purification cost, diluted 1,3-dioxolane was investigated. A suspension of dried cells (0.1 g, which was equal to 5% w/v) in 2 ml of diluted 95, 80, and 85% (v/v) 1,3-dioxolane was prepared, and PHB was extracted from the dried cells by incubating at 80°C for 6 h. The samples were mixed using a vortex mixer every 30 min.

The separation of PHB from the solution was performed by the addition of 3 volumes of water. Then, the purified PHB floating on the surface of the solution was separated by centrifugation at 4,620× *g* for 5 min at room temperature. The purified PHB was washed twice with water and dried overnight in an oven at 60°C and kept in a vacuum desiccator. The purified PHB was analyzed in terms of percentage purity and recovery by gas chromatography. Parallel extraction with undiluted 1,3-dioxolane was performed as a control experiment.

#### PHB extraction using a Soxhlet extractor

The extraction of PHB from dried cells and wet cells was performed using the Soxhlet extraction apparatus with 100 ml extractor body (Glassco Laboratory Equipments Pvt. Ltd., Haryana, India). The extraction was performed using a suspension of 5 g dried cells or 13.5 g wet cells in 100 ml 1,3-dioxolane. The extraction temperature was set above the boiling temperature at 100°C to attain multi-recycle of solvent extraction for 24 h. The separation of PHB after extraction was performed as described in the PHB Extraction Using Diluted 1,3-Dioxolane section.

#### PHB extraction using an ultrasonicator

To investigate the potential of using ultrasonication, which is one of the most widely used methods for mechanical cell disruption in the lab scale, the small-scale experiments were performed and compared with chemical cell disruption. Sonics vibra-cell VCX 130 (Sonics & Materials, Inc., USA) was used for ultrasonication. The sonicating unit contains a 3 × 137 mm (diameter × height) probe tip with a net power output of 130 W, frequency of 20 kHz, and amplitude of 40%. The suspension of 5% (w/v) wet cells in 2 ml of 1,3-dioxolane was prepared in borosilicate-glass test tubes. The duration subjected to ultrasonication was varied from 3, 6, and 10 min (Murugesan and Iyyaswami, 2017). The solution was centrifuged at 4,620× *g* for 5 min at room temperature. The separation of PHB after extraction was performed as described in the PHB Extraction Using Diluted 1,3-Dioxolane section. Parallel extraction with deionized water was performed as a control experiment.

#### PHB extraction using a shaking water bath

The effect of continuous mixing during the extraction process was compared with that of manual interval mixing every 30 min using a vortex mixer. The suspension of 5% (w/v) wet cells or dried cells in 2 ml of 1,3-dioxolane was prepared in screw-capped borosilicate-glass test tubes. The extraction was performed in a reciprocal shaking water bath (GFL 1086 shaking water bath, Gesellschaft für Labortechnik mbH, Burgwedel, Germany) by incubating at 80°C for 6 h, and the shaking speed was varied between 50 and 100 round per minute (rpm). The separation of PHB after extraction was performed as described in the PHB Extraction Using Diluted 1,3-Dioxolane section. Parallel extraction with 1,3-dioxolane as a control experiment was performed as described in the Comparision of PHB extraction efficiency between *C. necator* strain A-04 and *C. necator* H16 section.

### Scale-up PHB extraction process from 40 ml to 1,000 ml

The influence of the solid-to-solvent ratio on the efficiency of the extraction process was verified in 40 ml and then directly scaled up to 1,000 ml. First, the suspension of 2 g of dried cells or 5.4 g of wet cells in 40 ml of 1,3-dioxolane was prepared in 500 ml screw-capped DURAN^®^ borosilicate glass. The extraction was performed in a shaking water bath (SK-939XL shaking water bath, Amerex Instruments, Inc., CA, USA) by incubating at 80°C. The shaking speed was varied between 100 and 150 rpm, and the extraction time was varied from 3, 4, 5, and 6 h. Next, the solid-to-solvent ratio was varied from 2.5, 5, 7.5, and 10% (w/v). The extracting temperature was set at room temperature, (30°C) wet cell suspension in 40 ml of 1,3-dioxolane was kept in a shaking water bath with optimal shaking speed, and the extraction time was varied from 12, 24, and 36 h. Additionally, cell disruption with hot water by autoclaving prior to 1,3-dioxolane extraction was also investigated in both dried cells and wet cells. The autoclaved cells were subjected to 1,3-dioxolane extraction at room temperature with a shaking speed of 150 rpm, and the extraction time varied from 12, 24, and 36 h. Parallel extraction with 1,3-dioxolane in a shaking water bath at 80°C for 3 h was performed as a control experiment. The reduction of water addition in the PHB separation process was also studied by comparing 2 volumes and 3 volumes of water addition. Finally, the obtained extraction conditions were directly applied to 1,000 ml of 1,3-dioxolane in 5,000 ml screw-capped DURAN^®^ borosilicate glass. To reduce the demand for the purchase of new solvents, previously used 1,3-dioxolane was recycled in combination with new 1,3-dioxolane, and the ratio of reused 1,3-dioxolane and fresh 1,3-dioxolane was varied by 1:0, 1:1, and 1:2 (v/v) of the total volume. The percentage purity and recovery reused 1,3-dioxolane were compared with those obtained using new 1,3-dioxolane.

### Analytical methods

Cell growth was determined as the cell dry mass (CDM) by filtering 5 ml of the culture broth through pre-weighed cellulose nitrate membrane filters (pore size = 0.22 μm; Sartorius, Goettingen, Germany). The filters were dried at 80°C for 2 days and stored in desiccators to attain a constant weight. The net biomass was defined as the residual cell mass (RCM), which was calculated by subtracting the amount of PHB from the CDM. The PHB in dried cells was quantified by the whole cell methyl esterification with GC analysis (Braunegg et al., 1978). The resulting monomeric methyl esters were quantified by using a gas chromatograph (model CP3800, Varian Inc., Walnut Creek, CA, USA) using a Carbowax-PEG capillary column (0.25 μm df, 0.25 mm ID, 60 m length, Agilent Technologies Inc., Santa Clara, CA, USA). The internal standard was benzoic acid, and the external standard was PHB (Sigma-Aldrich Corp., St. Louis, MO, USA). The total reducing sugar concentration was determined using a 3,5-dinitrosalicylic acid (DNS) assay (Miller, 1959). The PHB purity (%) of the purified polymer and PHB recovery yield (%) were defined as follows (Riedel et al., 2013):

$$Purity\;\left(\%\right)=\frac{\left(g\right)PHB_{GC}\times 100}{\left(g\right)PHB_{recovery}}$$ (1)

$$Recovery\;\left(\%\right)=\frac{\left(g\right)PHB_{recovery}\times purity\left(\%\right)}{\left(g\right)PHB_{GC}}$$ (2)

where PHB_GC_ is the mass of PHB quantified by the whole cell methyl esterification with GC analysis and PHB_recovery_ is the mass of the extracted PHB after solvent evaporation.

### Analysis of mechanical properties of PHB films

The mechanical properties of PHA films were analyzed in accordance with the ASTM: D882-91 protocol (D882-91, 1991). The PHA films were prepared using conventional solvent-casting techniques and a glass tray (Pyrex, Corning Incorporated, NY, USA) as the casting surface (modified from Yoshie et al., 1995). The thickness of the thin polyester films was regulated by controlling the concentration of the polymer in chloroform (1% w/v) and the volume of the polymer solution. The thickness of the PHA films was 0.05 mm, which was confirmed using a caliper (Model 500–175: CD-12C, Mitutoyo Corporation, Kawasaki-shi, Kanagawa, Japan). A minimum of five film samples of 50 × 150 mm were cut and then aged for 1 week in a desiccator at ambient temperature to allow them to reach crystallization equilibrium before the analyses. The mechanical tests were conducted using a Universal Testing Machine (H10KM, Wuhan Huatian Electric Power Automation Co., Ltd., Wuhan, China) with a crosshead speed of 10 mm/min. The variables measured included the elongation at the break point (%), the stress at the maximal load (MPa), and Young’s modulus (MPa). Commercial PHB (Sigma-Aldrich Corp.) was also tested in the same conditions for comparison. The data represent the mean values for the minimum of five samples tested in the same conditions.

### Thermal analysis by differential scanning calorimetry

A 10 mg sample of PHB was encapsulated in an aluminum sample vessel and placed in the sample holding chamber of the differential scanning calorimetry (DSC) apparatus (204 F1 Phoenix, NETZSCH-Gerätebau GmbH Büro Rheinbach, Bonn, Germany). The previous thermal history of the sample was removed before the thermal analysis by heating the sample from ambient temperature to 230°C at 20°C/min. Next, the temperature was cooled down at 20°C/min to 50°C/min. The sample was then thermally cycled at 20°C/min to 230°C/min. The melting peak temperature, denoted by T_M_, was given by the intersection of the tangent to the furthest point of an endothermic peak and the extrapolated sample baseline. The glass transition temperature, denoted by T_g_, could be estimated by extrapolating the midpoint of the heat capacity difference between glassy and viscous states after heating the quenched sample.

### Molecular weight determination by gel permeation chromatography

The molecular weight was determined by gel permeation chromatography (GPC) (LC-10A; Shimadzu Corp., Kyoto, Japan) with a GPC K-804L column (8.0 mm ID × 300 mm L, Showa Denko K.K., Tokyo, Japan). The polymer was dissolved in 1% THF and filtered through a 0.45 μm membrane filter. A standard curve was determined for polystyrene in the same conditions for the molecular weight range of 5.00 × 10^2^ to 7.06 × 10^5^ Da.

### Data analysis

All the data presented in this manuscript are representative of the results of three independent experiments and are expressed as the mean values ± standard deviations (SDs). Analysis of variance (one-way ANOVA) followed by Duncan’s test for testing differences among means was conducted using SPSS version 22 (IBM Corp., Armonk, NY, USA). Differences were considered significant at *p* < 0.05.

## Results

### Comparison of PHB extraction efficiency between *C. necator* strain A-04 and *C. necator* H16 using 1,3-dioxolane

In the previous report, a water-miscible system was developed using 1,3-dioxolane and water to extract PHB from dried cells and wet cells at 2 ml and 40 ml scales. To make the scale-up process for this single step of PHB extraction and purification feasible, several factors needed to be investigated. Preliminarily, the representative Gram-negative bacterium harboring PHA granules used in this study were *C. necator* strain A-04 (Chanprateep et al., 2008) and *C. necator* H16. After batch cultivation in a 5 L bioreactor using 20 g/L of fructose with a C/N ratio of 200, *C. necator* strain A-04 containing 70.3 ± 2.9 %wt and *C. necator* H16 containing 67.2 ± 2.0 %wt were obtained. Wet cells and dried cells were prepared as described in the experimental section. In the 2 ml extraction scale, the efficiency of PHB recovery from dried cells of *C. necator* strain A-04 using 1,3-dioxolane was 91.6 ± 0.9% and the purity was 97.9 ± 0.2%, whereas that of *C. necator* H16 was 90.4 ± 1.3% and the purity was 97.2 ± 0.7%. The efficiency of PHB recovery from wet cells of *C. necator* strain A-04 using 1,3-dioxolane was 86.1 ± 1.8% and the purity was 97.9 ± 0.6%, whereas that of *C. necator* H16 was 86.8 ± 0.2% and the purity was 98.0 ± 0.4%. These results were comparable and confirmed that the single step extraction method developed in this study can be applied to industrial bacterial strain.

### The potential of using diluted 1,3-dioxolane for PHB extraction

**FIGURE 1:**
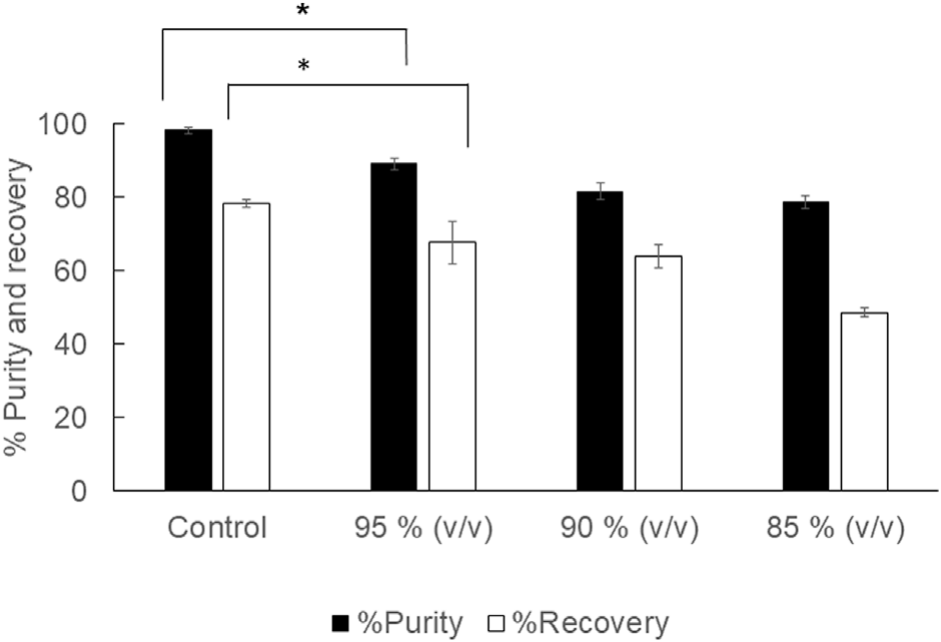
Effect of diluted 1,3-dioxolane varying from 95, 90, and 85% (v/v) on the % recovery and purity of the PHB extraction process. The control experiment was performed with undiluted 1,3-dioxolane. All the data are representative of the results of three independent experiments and are expressed as the mean values ± standard deviations (SDs). The asterisk indicates a significant difference (*p* < 0.05).

The aim of using diluted 1,3-dioxolane was to reduce the extraction cost incurred by the solvent price. 1,3-Dioxolane is a water-miscible solvent, but water acts as a plasticizer for PHB. Therefore, appropriate dilution was investigated in 2 ml of diluted 95, 90, 85% (v/v) 1,3-dioxolane. The results are shown in Figure 1. It was clearly observed that diluted 1,3-dioxolane possessed lower extraction efficiency than that in the control experiment. The recovery decreased from 78.3 ± 0.9% to 48.6 ± 1.3% as 1,3-dioxolane was diluted from 100 to 85% (v/v). The purity also decreased from 98.1 ± 0.7% to 78.8 ± 1.7%. Therefore, diluted 1,3-dioxolane is not applicable for PHB extraction from dried cells.

### PHB extraction using a Soxhlet extractor with 1,3-dioxolane

**FIGURE 2:**
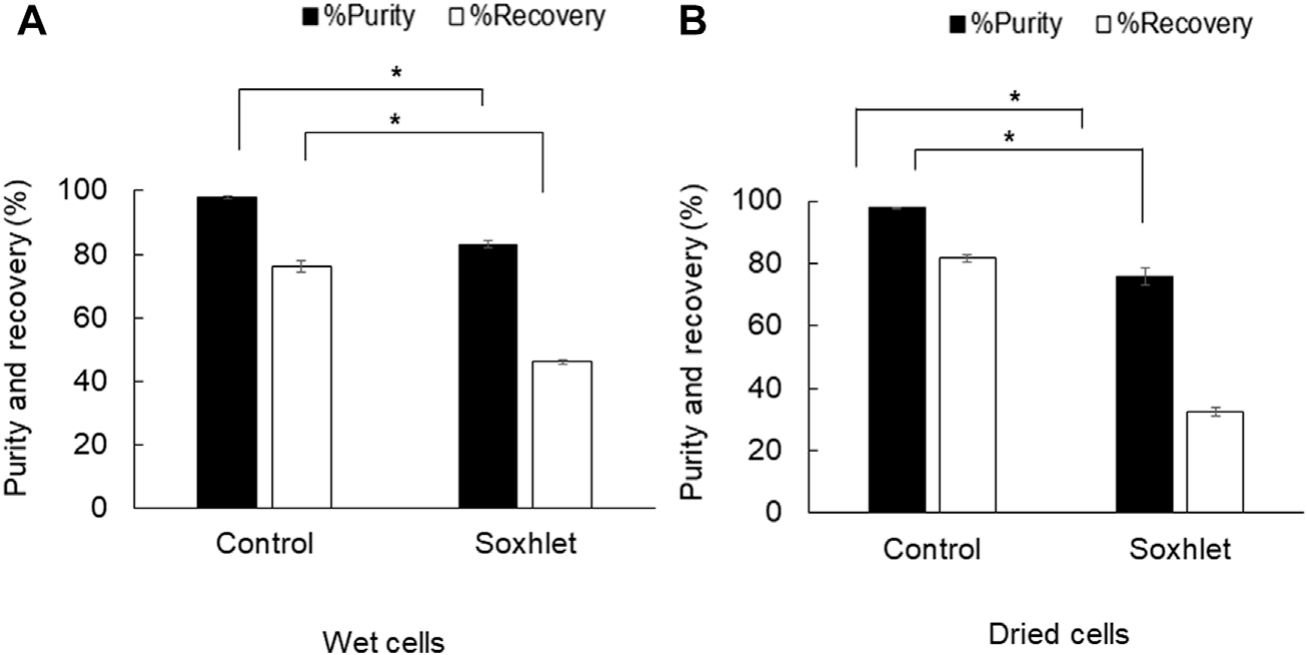
Efficiency of PHB extraction using a Soxhlet extractor with 1,3-dioxolane **(A)** from wet cells and **(B)** from dried cells. All the data are representative of the results of three independent experiments and are expressed as the mean values ± standard deviations (SDs). The asterisk indicates a significant difference (*p* < 0.05).

The objective of using a Soxhlet extractor in the PHB extraction process with 1,3-dioxolane was to attain continuous extraction with the increase in mass transfer rate based on refluxing and recycling of the fresh solvent that can also prevent saturation of solutes in the solvent. The Soxhlet extractor with 100 ml capacity was tested for PHB extraction from both dried cells and wet cells. The results shown in Figure 2 reveal that the Soxhlet extractor applied to wet cells gave the recovery of 46.0 ± 0.7% and purity of 83.2 ± 1.3%, whereas dried cells gave the recovery of 32.5 ± 1.2% and purity of 75.7 ± 2.9%. Thus, the Soxhlet extractor could not provide reasonable efficiency both in terms of recovery and purity.

### PHB extraction using an ultrasonicator

**FIGURE 3:**
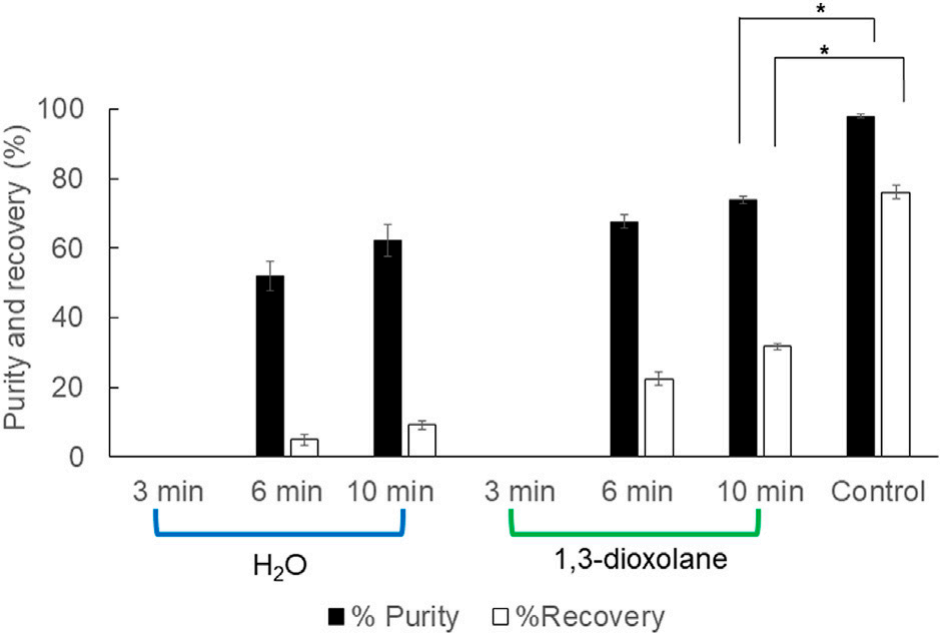
Efficiency of PHB extraction using ultrasonication in water and 1,3-dioxolane from wet cells. All the data are representative of the results of three independent experiments and are expressed as the mean values ± standard deviations (SDs). The asterisk indicates a significant difference (*p* < 0.05).

The ultrasonicator is a mechanical cell disruption instrument commonly used in the lab scale. Cell wall rupture prior to 1,3-dioxolane dissolution may increase PHB extraction efficiency. The operation time is significantly shorter than the control experiment. It was found that the ultrasonicator is applicable for PHB extraction from wet cells. The comparison between ultrasonication in water and 1,3-dioxolane showed that ultrasonication in 1,3-dioxolane gave better % purity and % recovery; however, they are significantly lower than those of control experiment. The extraction efficiency increased as the ultrasonication time increased up to 10 min both in water (recovery 9.2 ± 1.5% and purity 62.3 ± 4.7%) and 1,3-dioxolane (recovery 76.1 ± 1.8% and purity 97.9 ± 0.6%) (Figure 3). Considering the short extraction time by the aid of the mechanical cell disruption without heating requirement, it can be a promising strategy once the optimal conditions are verified.

### PHB extraction using a shaking water bath

**FIGURE 4:**
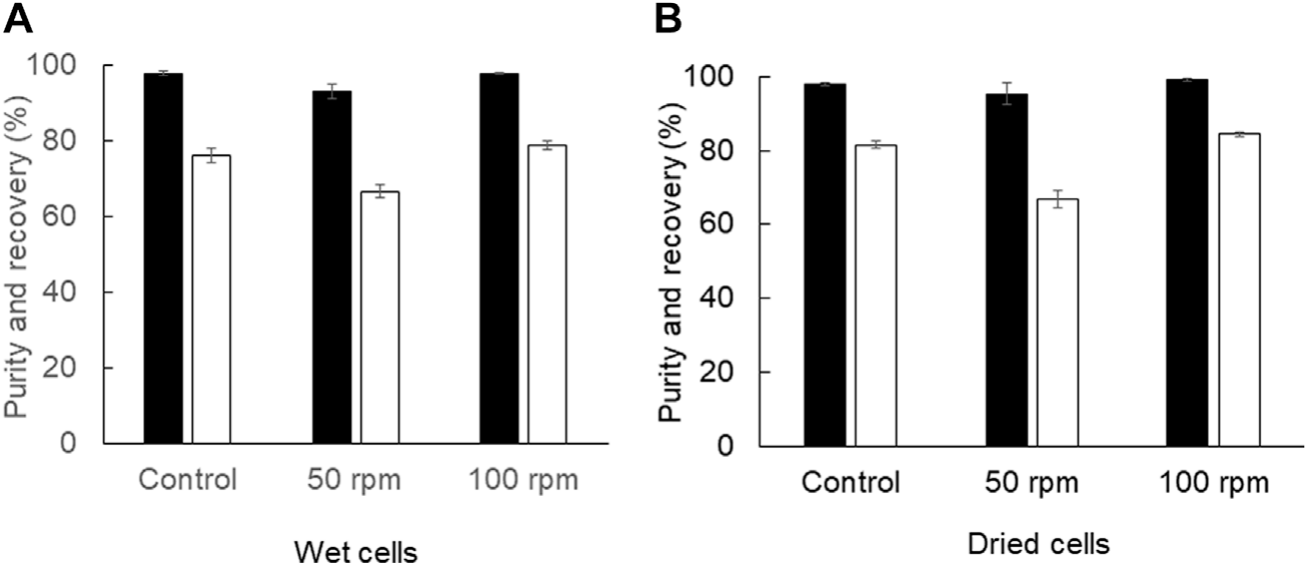
Efficiency of PHB extraction using a shaking water bath **(A)** from wet cells and **(B)** from dried cells. The shaking speed was varied from 50 and 100 rpm. The control experiment was performed with manual interval mixing every 30 min by a vortex mixer. All the data are representative of the results of three independent experiments and are expressed as the mean values ± standard deviations (SDs).

In addition to the Soxhlet extractor, the shaking water bath can provide continuous mixing to increase the mass transfer in the extraction process. The results showed that continuous mixing by a reciprocal shaking water bath at 100 rpm provided comparable PHB extraction efficiency to that of manual interval mixing every 30 min by using a vortex mixer (Figure 4). The recovery of 84.4 ± 0.7% and purity of 98.9 ± 0.5% from dried cells using a shaking water bath at 100 rpm were similar to the recovery of 81.6 ± 0.9% and purity of 97.9 ± 0.2% in a control experiment. The recovery of 78.9 ± 1.0% and purity of 97.9 ± 0.2% from wet cells using the shaking water bath at 100 rpm were similar to the recovery of 76.1 ± 1.8% and purity of 97.9 ± 0.5% in a control experiment. Altogether, the results are summarized in Table 1. The extraction using a shaking water bath was chosen in the direct scale-up process from 2 ml to 40 ml and 1,000 ml.

**TABLE 1:** Summary of results obtained from various types of PHB extraction methods from dried and wet cells using 1,3-dioxolane.

Scale (ml)	Cell type	Extraction condition	% recovery	% purity
2	Dried cells	Control: screw-capped tubes, 1,3-dioxolane, 80°C, 6 h, vortex every 30 min	81.6 ± 1.0	97.9 ± 0.3
		Screw-capped tubes, diluted 95% (v/v) 1,3-dioxolane, 80°C, 6 h, vortex every 30 min	67.6 ± 5.7	89.0 ± 1.6
		Soxhlet extractor, 1,3-dioxolane, 100°C, 24 h	32.5 ± 1.2	75.7 ± 2.9
		Shaking water bath, 1,3-dioxolane, 80°C, 6 h, 100 rpm	84.4 ± 0.7	98.9 ± 0.5
	Wet cells	Control: screw-capped test tubes, 1,3-dioxolane, 80°C, 6 h, vortex every 30 min	76.1 ± 1.8	97.9 ± 0.6
		Ultrasonication, 1,3-dioxolane, 10 min	31.8 ± 0.9	73.9 ± 1.1
		Soxhlet extractor, 1,3-dioxolane, 100°C, 24 h	46.0 ± 0.7	83.2 ± 1.3
		Shaking water bath, 1,3-dioxolane, 80°C, 6 h, 100 rpm	76.1 ± 1.8	97.9 ± 0.5
40	Dried cells	Control: screw-capped DURAN^®^ bottle, 1,3-dioxolane, 80°C, 6 h, vortex every 30 min	81.6 ± 1.0	97.9 ± 2.9
		Shaking water bath, 1,3-dioxolane, 80°C, 4 h, 150 rpm	96.6 ± 0.1	99.1 ± 0.6
		Shaking water bath, 1,3-dioxolane, 80°C, 3 h, 150 rpm, pre-cell disruption by autoclave	49.1 ± 0.5	94.7 ± 0.9
	Wet cells	Control: screw-capped DURAN^®^ bottle, 1,3-dioxolane, 80°C, 6 h, vortex every 30 min	76.7 ± 1.8	90.7 ± 0.1
		Shaking water bath, 1,3-dioxolane, 80°C, 4 h, 150 rpm	94.6 ± 4.8	97.0 ± 0.1
		Shaking water bath, 1,3-dioxolane, room temperature (30°C), 36 h, 150 rpm	93.5 ± 0.7	97.7 ± 1.3
		Shaking water bath, 1,3-dioxolane, room temperature (30°C), 36 h, 150 rpm, pre-cell disruption by autoclave	68.8 ± 1.0	97.6 ± 1.5
1,000	Dried cells	Shaking water bath, 1,3-dioxolane, 80°C, 4 h, 150 rpm	94.1 ± 0.4	99.3 ± 0.5
	Wet cells	Shaking water bath, 1,3-dioxolane, 80°C, 4 h, 150 rpm	92.6 ± 1.0	90.3 ± 1.5

### Scale-up PHB extraction from 40 ml to 1,000 ml

**FIGURE 5:**
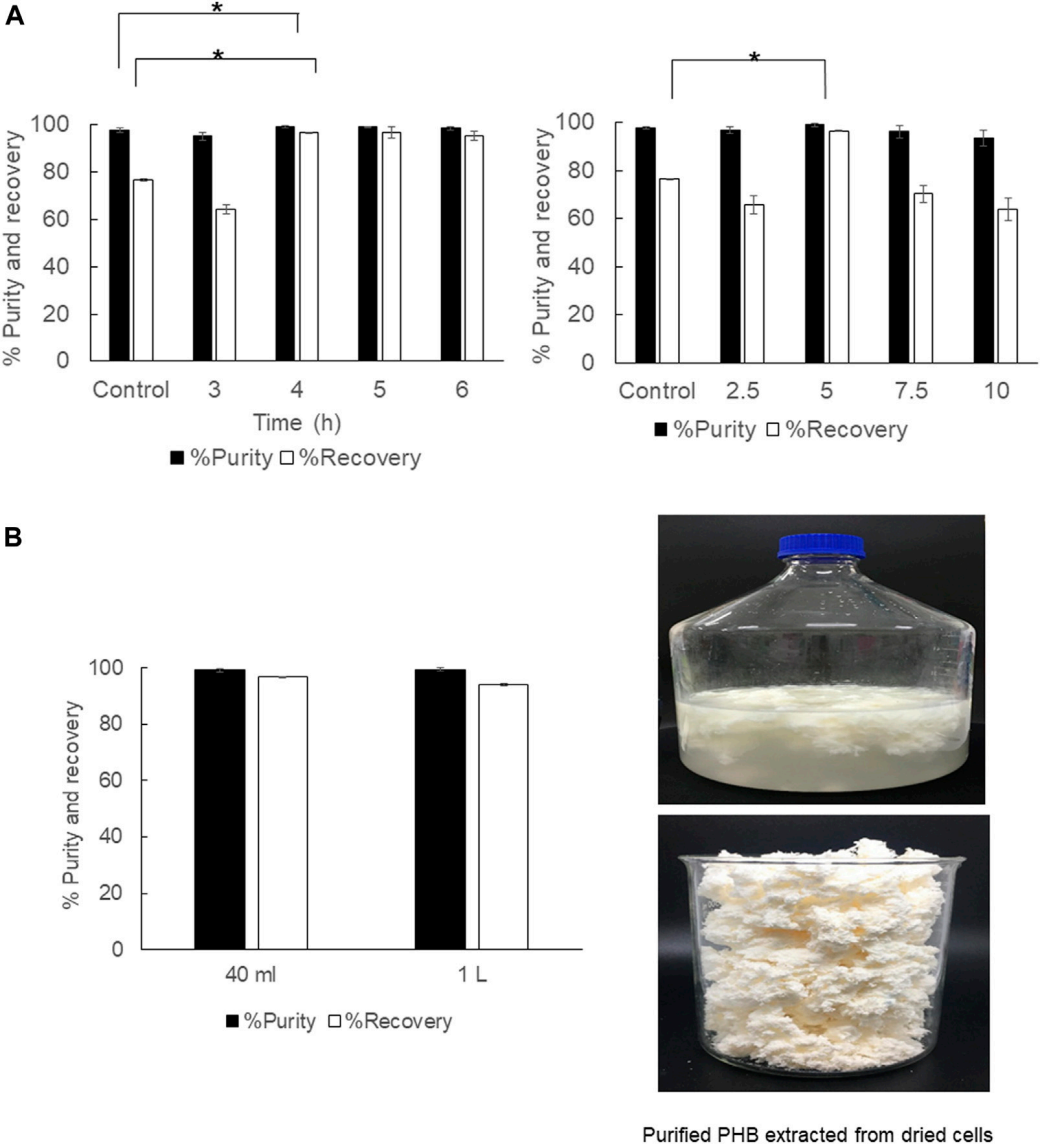
**(A)** Time courses of PHB extraction using a shaking water bath from dried cells at 150 rpm and the effect of the solid-to-solvent ratio varied from 2.5, 5, 7.5, and 10% (w/v). **(B)** The effect of direct scale-up from 40 ml to 1000 ml on the % purity and % recovery and the characteristics of purified PHB extracted from dried cells. All the data are representative of the results of three independent experiments and are expressed as the mean values ± standard deviations (SDs). The asterisk indicates a significant difference (*p* < 0.05).

The direct scale-up extraction process from 40 ml to 1,000 ml was subsequently performed in a shaking water bath by varying the shaking speed from 50, 100, and 150 rpm. In the 40 ml scale, the extraction time was varied from 3, 4, 5, and 6 h, and the solid-to-solvent ratio was varied from 2.5, 5, 7.5, and 10% (w/v). The results are shown in Table 2 and Figure 5. It is obviously seen that for dried cells the optimal extraction time, which was carefully verified every 1 h in this study, was 4 h. The solid-to-solvent ratio at 5% (w/v) gave the best results, the same as that obtained from 2 ml (Figure 5A). Finally, the optimal conditions for PHB extraction from dried cells, that is, extraction temperature at 80°C, shaking speed at 150 rpm, extraction time for 4 h, and the solid-to-solvent ratio of 5% (w/v), were applied in the 1,000 ml extraction process. The recovery of 94.1 ± 0.4% and purity of 99.3 ± 0.5% were obtained. It was concluded the % recovery was insignificantly reduced when compared with 96.6 ± 2.4% obtained from the 40 ml extraction scale. However, the scale-up process from 40 ml to 1,000 ml did not affect % purity. The purified PHB obtained from this simple extraction process is shown in Figure 5. The PHB powder showed off-white color (Figure 5B). The developed extraction process used only 1,3-dioxolane and the product separation used only water. Dried cells also have some advantages over wet cells because it can be stored at room temperature until use.

**TABLE 2:** Effect of shaking speed of the water bath on PHB extraction from dried and wet cells using 1,3-dioxolane.

Shaking speed (rpm)	Scale (ml)	% recovery	% purity
50	Dried cells	66.8 ± 2.2	95.3 ± 2.2
	2 ml		
	40 ml	67.2 ± 0.4	97.7 ± 0.7
100	Dried cells	83.9 ± 1.4	99.0 ± 0.4
	2 ml	85.5 ± 0.2	99.0 ± 0.2
	40 ml		
	Wet cells	80.6 ± 2.0	97.1 ± 0.4
	40 ml		
150	Dried cells	96.6 ± 0.1	99.1 ± 0.6
	40 ml		
	1,000 ml	94.1 ± 0.4	99.3 ± 0.5
	Wet cells	94.6 ± 4.8	97.0 ± 0.1
	40 ml		
	1,000 ml	92.6 ± 1.0	90.3 ± 1.5
Control	Dried cells	81.6 ± 1.0	97.9 ± 2.9
	40 ml		
	Wet cells	76.7 ± 1.8	97.9 ± 0.1
	40 ml		

**FIGURE 6:**
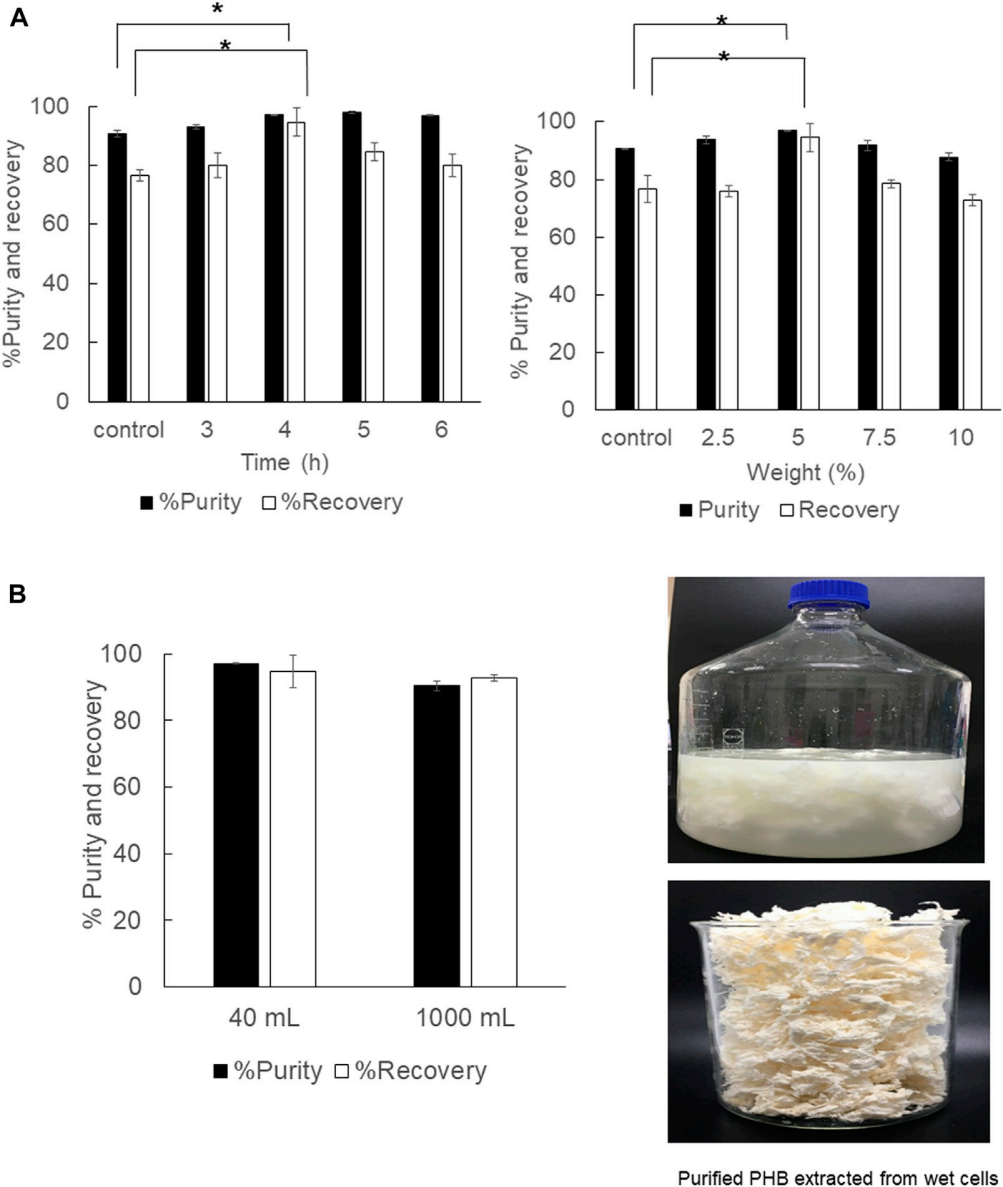
**(A)** Time courses of PHB extraction using a shaking water bath from wet cells at 150 rpm and the effect of the solid-to-solvent ratio varied from 2.5, 5, 7.5, and 10% (w/v). **(B)** The effect of direct scale-up from 40 ml to 1000 ml on the % purity and % recovery and the characteristics of purified PHB extracted from wet cells. All the data are representative of the results of three independent experiments and are expressed as the mean values ± standard deviations (SDs). The asterisk indicates a significant difference (*p* < 0.05).

The optimal extraction time and solid-to-solvent ratio for PHB extraction from wet cells were verified in parallel. The results are shown in Figure 6. The extraction time for wet cells was reduced from 6 h to 4 h, and the continuous shaking enhanced the % recovery and % purity significantly when compared with manually mixing every 30 min by using a vortex mixer. The solid-to-solvent ratio for PHB extraction from wet cells was also the same at 5% (w/v) as those obtained from dried cells (Figure 6A). Finally, the optimal conditions for PHB extraction from dried cells, extraction temperature at 80°C, shaking speed at 150 rpm, extraction time for 4 h, and the solid-to-solvent ratio of 5% (w/v) were applied in the 1,000 ml extraction process in a 5,000 ml screw-capped DURAN^®^ borosilicate glass. The recovery insignificantly decreased from 94.6 ± 4.8% to 92.6 ± 1.0%, and the purity slightly decreased from 97.0 ± 0.1 to 90.3 ± 1.5%. The characteristics of the purified PHB extracted from wet cells are shown in Figure 6B. The white puffy powder immediately floated on the surface of the 1,3-dioxolane solution after adding 3 volumes of water. Although PHB extraction from wet cells showed lower efficiency than that of dried cells, wet cells have potentials for further development of the extraction process due to their cell lysis readiness and safe time and heating energy for dried cells preparation.

### PHB extraction at room temperature in the 40 ml extraction scale

**FIGURE 7:**
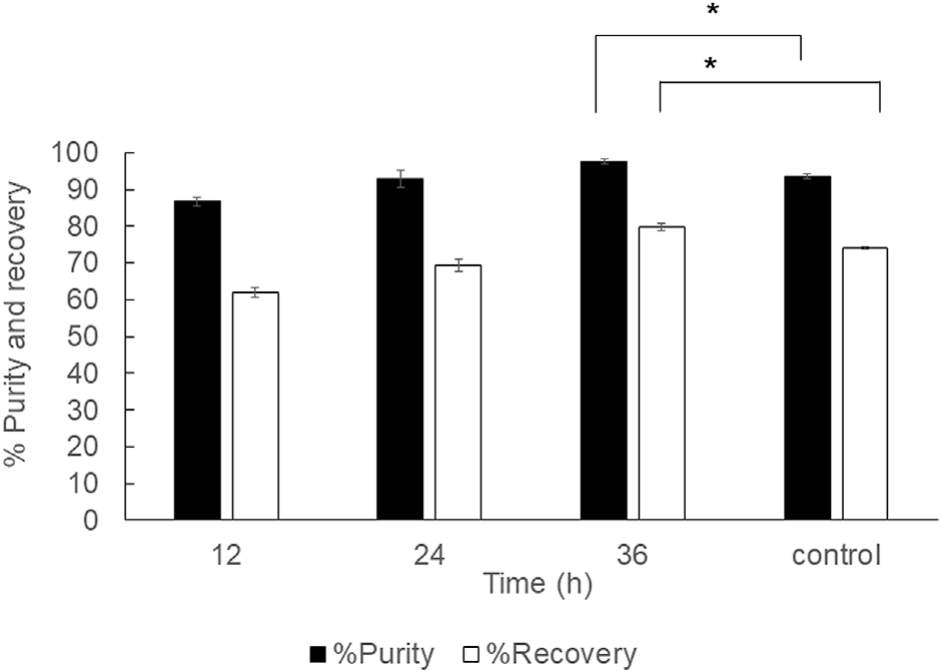
Time courses of PHB extraction using a reciprocal shaking water bath from wet cells at 150 rpm under room temperature. All the data are representative of the results of three independent experiments and are expressed as the mean values ± standard deviations (SDs). The asterisk indicates a significant difference (*p* < 0.05).

In the previous report, the 2 ml PHB extraction at room temperature was performed at room temperature (Yabueng and Napathorn, 2018). Here, the extension study was performed to verify the optimal conditions for PHB extraction under room temperature from wet cells at the 40 ml scale using a shaking water bath with an optimal shaking speed at 150 rpm and varied extraction time from 12, 24, and 36 h. The results were compared with the control experiments performed with the conditions reported previously (Yabueng and Napathorn, 2018). The results are shown in Figure 7. The continuous shaking by a shaking water bath clearly enhanced the recovery and purity from 74.1 ± 0.4% and 93.5 ± 0.7% to 79.8 ± 1.0% and 97.7 ± 1.3%, respectively. The PHB extraction under room temperature may not be applicable in the industrial scale due to long extraction time.

### Effect of cell disruption with hot water by autoclaving prior to 1,3-dioxolane extraction

**FIGURE 8:**
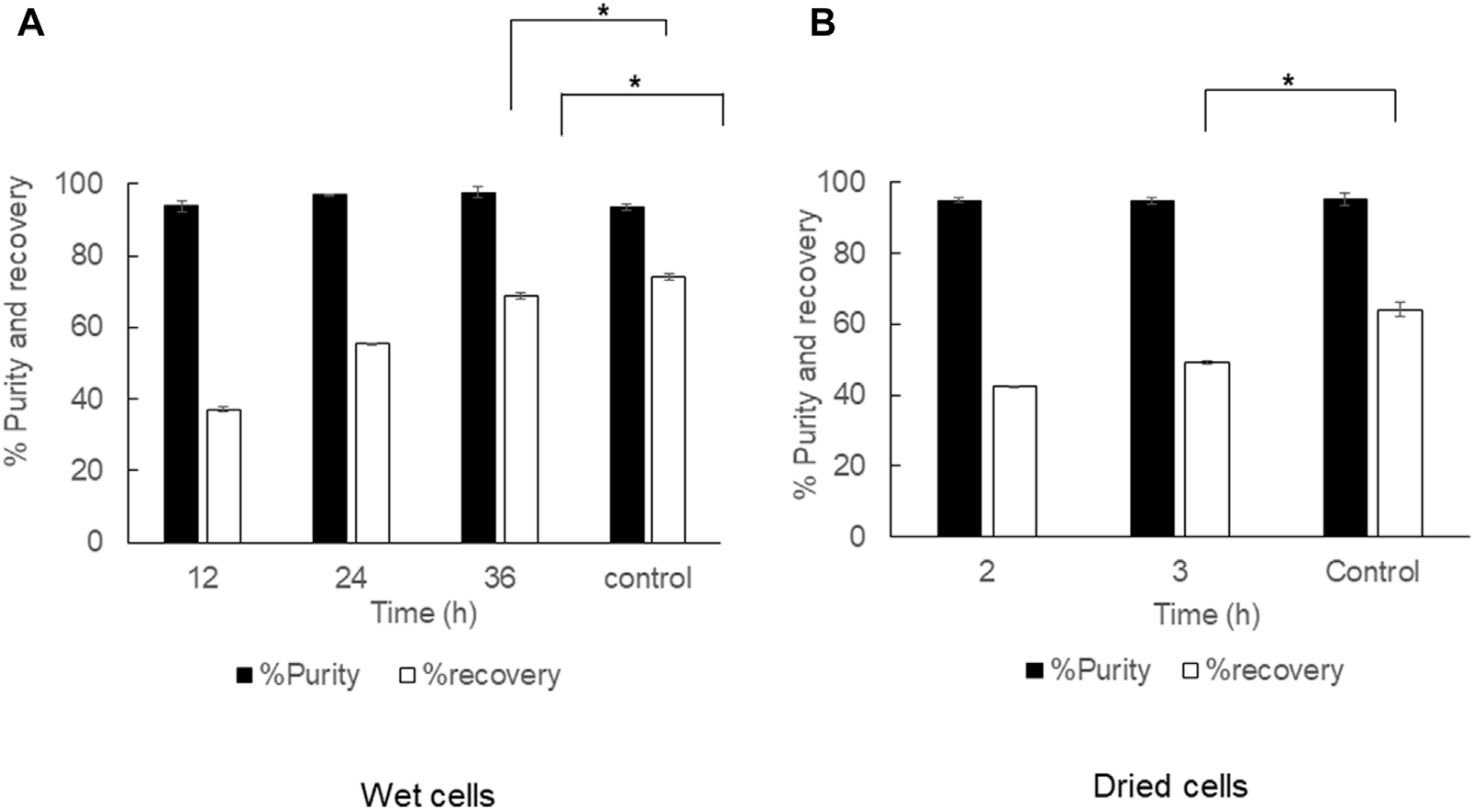
Time courses of PHB extraction from cell rupture by steam explosion prior to 1,3-dioxolane extraction. **(A)** Wet cells at 150 rpm under room temperature and **(B)** dried cells at 150 rpm under 80°C. All the data are representative of the results of three independent experiments and are expressed as the mean values ± standard deviations (SDs). The asterisk indicates a significant difference (*p* < 0.05).

The pretreatment of cell disruption with hot water by autoclaving prior to 1,3-dioxolane extraction was performed in both dried cells and wet cells under different conditions. The autoclaved dried cells were subjected to 1,3-dioxolane extraction at 80°C with a shaking speed of 150 rpm, and the extraction time was varied from 2 and 3 h. Parallel extraction of dried cells with 1,3-dioxolane in a shaking water bath at 80°C for 3 h was performed as a control experiment (Figure 8). The cell disruption with hot water by autoclaving did not influence the purity but significantly reduced the recovery from 64.2 ± 2.0% to 49.1 ± 0.5%. The effect of autoclaving wet cells prior to 1,3-dioxolane extraction under room temperature increased the purity from 93.5 ± 3.4% to 97.6 ± 1.5%, but the recovery significantly decreased from 74.1 ± 1.0% to 68.8 ± 1.0%. Concerning the cost incurred by the autoclave and the time consumed, cell disruption by autoclaving was not suitable for further development.

### Effect of reducing water addition in the solid–liquid separation process

In the previous study, the effect of water addition on the PHB precipitation in 1,3-dioxolane was investigated in the 2 ml extraction scale. Here, in the 1,000 ml extraction scale, the effect of reducing water addition in a PHB solid–liquid separation process was studied and compared % purity of PHB obtained from adding 3 volumes of water (98.1 ± 0.4%) and 2 volumes of water (99.0 ± 0.5%). The results revealed that reducing water addition to 2 volumes of water did not significantly change the % purity of PHB; thus, the use of water could be reduced.

### Effect of using reused 1,3-dioxolane for PHB extraction

To reduce the demand for the purchase of new solvents, the used 1,3-dioxolane was recycled in combination with new 1,3-dioxolane, and the ratio of reused 1,3-dioxolane and fresh 1,3-dioxolane was varied by 1:0, 1:1, and 1:2 (v/v) of the total volume (Table 3). The boiling temperature of 1,3-dioxolane is 76°C and water is 100°C. In fact, recycling of 1,3-dioxolane was tested using a vacuum rotary evaporator, but it was found that separation of these two solvents by boiling was difficult because of the limitation of the vacuum rotary evaporator, which may require more than 20°C differences in boiling points. Therefore, the used 1,3-dioxolane was directly mixed with the new 1,3-dioxolane, and the ratio of 1:2 gave the recovery of 42.0 ± 1.2% and purity of 69.4 ± 2.4% from dried cells, whereas the recovery and purity were 57.4 ± 2.0% and 81.3 ± 1.0% from wet cells. Therefore, recycling of 1,3-dioxolane requires further development.

**TABLE 3:** Effect of reused 1,3-dioxolane on % recovery and % purity of PHB.

Cell types	Ratio of used and new 1,3-dioxolane	% recovery	% purity
Dried cells	1:0	0	0
	1:1	0	0
	1:2	42.0 ± 1.2	69.4 ± 2.4
Wet cells	1:0	0	0
	1:1	0	0
	1:2	57.4 ± 2.0	81.3 ± 1.0

### Effect of 1,3-dioxolane extraction on the molecular weight distribution and thermal and mechanical properties

Table 4 shows the molecular weight distribution and thermal and mechanical properties of purified PHB obtained from 1,3-dioxolane extraction during the scale-up process. The molecular weight distribution of purified PHB was not influenced by 1,3-dioxolane extraction at 80°C and 150 rpm of shaking speed for 4 h from dried cells and wet cells. The direct scale-up process from 40 ml to 1,000 ml also showed comparable values of M_W_, M_N_, and PDI, as well as T_M_, T_g_, and mechanical properties. Interestingly, it was found that PHB extraction from wet cells using 1,3-dioxolane at room temperature and 150 rpm of shaking speed for 36 h gave all properties similar to those obtained from 1,3-dioxolane extraction at 80°C and 150 rpm of shaking speed for 4 h. Therefore, heating systems can be omitted to attain safety operation.

**TABLE 4:** Effect of 1,3-dioxolane extraction on the molecular weight distribution and thermal and mechanical properties.

Condition	Purity	Recovery	M_W_	M_N_	PDI	T_M_	Tg	Young’s modulus	Stress at maximum load	Elongation
	(%)	(%)	(x 10^5^)	(x 10^5^)		(°C)	(°C)	(MPa)	(MPa)	(%)
Commercial PHB (CAS 26063–00–3 Batch-02925CB, Sigma-Aldrich)	—	—	4.3	2.3	1.9	172.0		3187.3	40.3	1.4
Chloroform (80°C, manual vortex every 30 min, 6 h)										
Dried cells (40 ml)	98.8 ± 1.7	94.5 ± 1.0	3.5	2.2	1.6	175.9	3.6	2315.0	14.6	1.7
1,3-Dioxolane (80°C, 150 rpm of shaking speed, 4 h)										
Dried cells (1,000 ml)	99.3 ± 0.5	94.1 ± 0.4	3.7	2.4	1.5	177.1	4.2	2515.8	22.2	2.2
Wet cells (1,000 ml)	90.3 ± 1.5	92.6 ± 1.0	3.5	2.6	1.3	177.5	-3.4	2629.6	21.0	4.2
1,3-Dioxolane (RT, 150 rpm of shaking speed, 36 h)										
Wet cells (40 ml)	97.7 ± 1.3	93.5 ± 0.7	3.1	2.7	1.1	178.2	-3.2	2874.7	22.5	2.2
Autoclaved wet cells (40 ml)	97.6 ± 1.5	68.8 ± 1.0	2.3	1.9	1.2	177.0	-2.3	2904.0	30.6	1.8

## Discussion

The objective of this study was to investigate the important factors influencing the PHB extraction using 1,3-dioxolane and scale up the extraction process from 2 ml to 1,000 ml. To start this study, the control experiment with the condition reported previously was applied to *C. necator* strain A-04 and compared with that of the industrial strain *C. necator* H16. The results confirmed that the method established in this study is reliable. Next, several factors were evaluated one factor at a time. First, the potential use of diluted 1,3-dioxolane, which is a water-miscible solvent, was tested. In fact, 1,3-dioxolane is classified into green solvent categories (Moscoso et al., 2014; Winterton, 2021). However, the use of 1,3-dioxolane is not advantageous economically because the cost is unduly high. Therefore, 1,3-dioxolane was diluted from 100, 95, 90, and 85% (v/v) and used to extract PHB from dried cells. It was found that all diluted 1,3-dioxolane significantly lowered the recovery yield and purity of PHB compared with those of concentrated 1,3-dioxolane (Figure 1). One possibility is that the addition of water into 1,3-dioxolane increases the boiling temperature of the diluted solvent. The boiling temperature of 1,3-dioxolane is 76°C, and the boiling temperature of water is 100°C. Therefore, the boiling temperature of the diluted solvent is elevated above 76°C. Hence, the extraction temperature of 80°C for diluted 1,3-dioxolane was not sufficient, resulting in low extraction performance. The extraction temperature is one of the important factors in the PHB solvent extraction process (Fiorese et al., 2009; Samorì et al., 2015; Yabueng and Napathorn, 2018). Several studies also reported that the extraction temperature should be above the boiling temperature of the mixture (Samorì et al., 2015; Onyebuchi and Kavaz, 2020). Their results also disclosed that the extraction temperature had an effect on the recovery yield. Increasing extraction temperature decreases the viscosity of the solvent, which consequently improves the penetration of the solvent into the sample matrix (Mustafa and Turner, 2011; Getachew et al., 2022). This phenomenon increases the diffusion of the analyte into the solvent and improves the extraction yield (Naviglio et al., 2019). Increasing temperature could facilitate the extraction yield by increasing both the solubility and mass transfer rates. However, very high temperatures could have a negative impact on the yield. At very high temperatures, the produced PHB could be further decomposed into smaller molecules or organic acids that consequently decrease the extraction yield (Aramvash et al., 2018; Alfano et al., 2021).

Next, a Soxhlet extractor with 100 ml capacity was applied to increase the heat and mass transfer rates based on refluxing and recycling of fresh 1,3-dioxolane for PHB extraction. Soxhlet extraction is still used as a reference method in the lab scale in official methods of the United States Environmental Protection Agency (USEPA), the Association of Official Analytical Collaboration (AOAC), and British standards (Barbero et al., 2022). The authors expected that recovery yield (%) can be improved under the recycling of the fresh solvent by Soxhlet extractor. The diffusion rate of PHB dissolution from dried or wet cells to the fresh solvent could be enhanced because continuous repetition of the extraction was achieved. The temperature at the solvent boiling flask was set above the boiling temperature at 100°C to attain multi-recycle of solvent extraction for 24 h. However, the results revealed that the recovery yield and purity of PHB extraction from both dried and wet cells were lower than those of the control experiment. By refluxing the solvent through the thimble using a condenser, the solvent temperature at the extractor was cooled down, which, in turn, reduced the extraction efficiency. Nevertheless, it was reported on the use of a Soxhlet extractor with the purpose of reducing the volume of the solvent used and compared with the direct solvent extraction (Koller et al., 2013). It was concluded that the PHA recovery from bacterial cells using a Soxhlet extractor with some PHB dissolution solvents (such as acetone and chlorinated solvents) was higher than that of direct solvent extraction (Jiang et al., 2006; Lupescu et al., 2016; Pagliano et al., 2021). The different conclusion may be attributed to the different size in Soxhlet capacity used because it was observed in this study that the extraction time in the thimble was quite short. Conversely, the use of a Soxhlet extractor to extract PHB with 1,3-dioxolane requires a heating system for the extractor unit as PHB-enriched solvents can form a stable gel upon cooling (Riedel et al., 2013; Aramvash et al., 2018).

Subsequently, the effect of mechanical cell disruption by ultrasonication without heating requirement was investigated and compared to the solvent medium between water and 1,3-dioxolane. The superior advantage of this developed procedure is a very short operation time. The extraction efficiency increased as the ultrasonication time increased from 3 to 10 min, and ultrasonication in 1,3-dioxolane gave the recovery of 76.1 ± 1.8% and purity of 97.9 ± 0.6% (Figure 3). The % purity obtained in this study was higher than 78.37% produced by ultrasonication of crude broth at 4 kHz for 5 min (Murugesan and Iyyasamy, 2017). Ultrasonication with the combination of nonionic surfactants at 3 pH with the addition of 0.1 M ammonium chloride in the mixed surfactant system at a reduced cloud point temperature of 33°C gave a relatively higher recovery of 84.4% than our results but gave a PHB purity of 92.5%, which was lower than that in our study (Murugesan and Iyyasamy, 2017). Martínez-Herrera et al. (2020) also reported the application of ultrasonic and solvent-free extraction protocol as a part of green trend for the PHB production process, but the recovery yield and purity were lower than those of this study. It is clearly demonstrated that PHB extraction using ultrasonication in 1,3-dioxolane is a promising strategy. The optimal conditions will be investigated in the future study.

Afterward, the manual vortex every 30 min using a vortex mixer was replaced with the shaking water bath to increase the mass transfer rate by continuous shaking, which further improves the transfer equilibrium. Preliminary investigation was performed in the 2 ml scale. It was found that continuous mixing by the shaking water bath at 100 rpm provided comparable PHB extraction efficiency to that of manual interval mixing every 30 min by a vortex mixer. In the 2 ml scale, the recovery of 84.4 ± 0.7% and purity of 98.9 ± 0.5% were obtained from dried cells and the recovery of 78.9 ± 1.0% and purity of 97.9 ± 0.2% were obtained from wet cells. Next, the direct scale-up experiment to 40 ml was conducted in screw-capped DURAN^®^ bottles. The shaking speed was varied from 50, 100, and 150 rpm, and the extraction time was varied from 3, 4, 5, and 6 h. The solid-to-solvent ratio was also varied from 2.5, 5, 7.5, and 10% (w/v) because the geometry of screw-capped glass test tubes was different from that of screw-capped DURAN^®^ bottles (Table 2 and Figure 5). In the 40 ml scale, the optimal conditions were 5% (w/v) solid-to-solvent ratio, 150 rpm of reciprocal shaking speed, and 4 h extraction time. The recovery of 96.6 ± 0.1% and purity of 99.1 ± 0.6% were obtained from dried cells and the recovery of 94.6 ± 4.8% and purity of 97.0 ± 0.1% were obtained from wet cells.

Finally, in the 1,000 ml scale, the recovery of 94.1 ± 0.4% and purity of 0.3 ± 0.5% were obtained from dried cells and the recovery of 92.6 ± 1.0% and purity of 90.3 ± 1.5% were obtained from wet cells. The % recovery from dried cells and wet cells did not significantly change in the scale-up process. On one hand, the % purity from dried cells also did not significantly change in the scale-up scheme. On the other hand, the % purity from wet cells slightly decreased when scaled up from 40 ml to 1,000 ml. Therefore, our single step extraction process provided better efficiency than that reported in other reports (Manangan and Shawaphun, 2010; Fei et al., 2016; Aramvash et al., 2018; Mongili et al., 2021). Furthermore, solid–liquid separation by addition of water into PHB solubilized in 1,3-dioxolane provides PHB powder with off-white color. The simple operation facilitates the environmental constant across scales to achieve comparable % recovery and % purity. However, heating 1,3-dioxolane above its boiling temperature was our concern because of flammability and formation of explosive vapor. In fact, 1,3-dioxolane does not form flammable gases in contact with water and has no pyrophoric properties. The self-ignition temperature of 1,3-dioxolane is 250°C. Based on these data, 1,3-dioxolane is classified as a flammable liquid (cat. 2) (European Chemicals Agency, 2022). Therefore, the extraction temperature was tested at room temperature (30°C) using the reciprocal shaking water bath at 150 rpm for 36 h, and the recovery of 93.5 ± 0.7% and purity of 97.7 ± 1.3% were obtained. To reduce the extraction time, cell rupture by steam explosion using an autoclave prior to 1,3-dioxolane extraction was evaluated and found that both autoclaved dried cells and wet cells gave relatively low % recovery. It should be noted that the long extraction time of 36 h at room temperature (30°C) and short extraction time of 4 h at 80°C did not show any effect on molecular weight distribution, thermal properties, and mechanical properties. The superior advantage of the established extraction method is that it can be applied to both dried cells and wet cells under the same conditions. Comparable studies have reported that ethyl acetate has been successfully used as a solvent for the extraction of poly(3-hydroxybutyrate-co-3-hydroxyhexanoate) (P3HB-co-3HHx) containing >15 mol% of HHx, resulting in a recovery yield of 99 wt% at 100°C (Riedel et al., 2013). Additionally, this study indicated the necessity to perform the extraction on dried cells since the presence of water had a double effect on ester hydrolysis and the reduction of the solvating power (Alfano et al., 2021).

## Conclusion

The efficiency of extraction methods depends on the critical parameters, the chemical properties of solvents, and the nature of compounds to be extracted. The best results obtained by different techniques are summarized in Table 1.

1) According to this study, PHB extraction using a Soxhlet extractor with 1,3-dioxolane exhibited poor PHB extraction performance from both dried cells (recovery yield of 32.5 ± 1.2% and purity of 75.7 ± 2.9%) and wet cells (recovery yield of 46.0 ± 0.7% and purity of 83.2 ± 1.3%) due to insufficient solvent temperature and short extraction time in the extractor unit.
2) Ultrasonication showed a similar recovery yield of 31.8 ± 0.9% with a purity of 73.9 ± 1.1%. It should be noted that the operation time was only 10 min under room temperature. Improvement in extraction efficiency using ultrasonication will be carried out in the future.
3) The shaking water bath was used to scale up the extraction process from 2 ml, 40 ml, and 1,000 ml. Several parameters including shaking speed, extraction time, solid-to-liquid ratio, extraction temperature, reused 1,3-dioxolane, and pre-cell disruption using autoclave were investigated. In the 1,000 ml extraction scale using the shaking water bath, 80°C, 4 h, and 150 rpm gave the best results from both wet cells and dried cells.
4) Additionally, PHB extraction from wet cells using 1,3-dioxolane using a shaking water bath at room temperature gave an interesting recovery yield of 93.5 ± 0.7% with a purity of 97.7 ± 1.3%. The primary advantage of solvent extraction at room temperature without heating systems is the safe operation, without a health risk caused by flammability. The future direction of this study is to pave the way for a low-cost and safe extraction platform (American Society for Testing and Materials, 1993).

## Data Availability

The raw data supporting the conclusions of this article will be made available by the authors, without undue reservation.
